# Plant traits and community composition drive the assembly processes of abundant and rare fungi across deserts

**DOI:** 10.3389/fmicb.2022.996305

**Published:** 2022-09-28

**Authors:** Jianming Wang, Mengjun Qu, Yin Wang, Nianpeng He, Jingwen Li

**Affiliations:** ^1^School of Ecology Nature Conservation, Beijing Forestry University, Beijing, China; ^2^Key Laboratory of Ecosystem Network Observation and Modeling, Institute of Geographic Sciences and Natural Resources Research, Chinese Academy of Sciences, Beijing, China

**Keywords:** deserts, community assembly, abundant and rare fungi, heterogeneous selection and dispersal limitation, plant functional traits and composition

## Abstract

The difference in community assembly mechanisms between rare and abundant fungi in deserts remains unknown. Hence, we compared the distribution patterns of abundant and rare fungi, and assessed the factors driving their assembly mechanisms across major vegetation types (shrubby desert, semi-shrubby and dwarf semi-shrubby desert, dwarf semi-arboreous desert, and shrubby steppe desert) of Chinese deserts. We assessed abundant and rare fungal subcommunities base on the sequencing data of fungal ITS data. Abundant fungal assembly was more affected by neutral processes than the rare. Null model and VPA analysis indicated that heterogeneous selection dominated rare sub-communities, whereas abundant fungal assembly was mainly determined by heterogeneous selection, dispersal limitation and other, unknown processes together. As a result, abundant sub-communities exhibited a higher species turnover rate than the rare. Hierarchical partitioning analysis indicated that soil conditions and plant attributes drove the assembly processes of abundant and rare fungi, respectively. Meanwhile, the relative strength of different assembly processes differed significantly among four vegetation types. In addition, we found that plant functional traits and composition played more critical roles in shaping the assembly processes of rare fungi than those of abundant fungi. Taken together, our findings collectively suggest that rare and abundant fungi exhibit differential ecological patterns that are driven by distinct assembly processes in deserts. We emphasize that the assembly processes of abundant and rare fungi are dependent on different abiotic and biotic factors in desert ecosystems.

## Introduction

Deserts cover 33% of the global land area (Cherlet et al., 2018), and encompass one of the most extreme environments on the earth (Alsharif et al., 2020). Notably, desert ecosystems are suffering climate changes and extreme weather events (Easterling et al., 2000; Dai, 2013), which may result in substantial alteration in ecosystem structure and function. Soil microbes play crucial roles in mediating ecosystem key processes and functioning, such as nutrient and material cycles (Mooshammer et al., 2014; De Sosa et al., 2018). Moreover, soil microbial communities are primarily dominated by a few abundant species, while a mass of other species (“rare biosphere”) have extremely low abundance (Pedrós-Alió, 2012; Jia et al., 2018; Egidi et al., 2019). Previous studies have reported that abundant and rare microbes play important but distinct roles in ecosystem functioning (Pedrós-Alió, 2012; Zhang Y. et al., 2019). Interestingly, owing to the difference in functional traits, rare and abundant microbial communities are subjected to divergent controlling mechanisms (Liu et al., 2015; Gao et al., 2020), thereby exhibiting quite diverse distribution patterns (Jiao and Lu, 2020a; Wang et al., 2021a). Comparing the biogeography and assembly mechanisms of rare and abundant microbes in deserts is critical for understanding how dryland ecosystems respond to environmental changes.

To date, biogeographical studies on abundant and rare bacteria have been extensively conducted in diverse environments (Jiao and Lu, 2020b; Wang et al., 2021b), whereas only a handful of studies have focused on abundant and rare fungi at the large scale (Jiao and Lu, 2020a; Wan et al., 2021). Compared with bacteria, fungi have a larger body size, and are more easily affected by long-term dispersal limitations (Powell et al., 2015). Moreover, fungi can decompose complex molecules from plant litter that are inaccessible to almost bacteria (Boer et al., 2005; Romaní et al., 2006), and can obtain and coordinate resources through generating hyphal networks in a nutrient-limited environment (Peay et al., 2016; Jiang et al., 2021), which may induce a difference in the distribution patterns and assembly mechanisms between soil bacteria and fungi. Hence, a targeted comparison of the assembly processes between rare and abundant fungi may shed novel insights into microbial assembly mechanisms. Previous studies have explored the major factors determining species richness and composition of abundant and rare bacteria in deserts (Wang et al., 2021b). Moreover, desert soil fungal diversity and composition have been well explored (Gonçalves et al., 2016; Santiago et al., 2018; Gómez-Silva et al., 2019). However, to date the difference in biogeography and assembly mechanisms between abundant and rare fungi across deserts has been barely elucidated.

It is generally believed that stochastic (e.g., homogenizing dispersal and dispersal limitation) and deterministic processes (e.g., homogeneous and heterogeneous selection) work together to drive soil microbial communities (Stegen et al., 2013; Dini-Andreote et al., 2015; Evans et al., 2017), whereas their relative roles vary between abundant and rare sub-communities (Jiang et al., 2019; Ji et al., 2020). For example, abundant fungal sub-communities are mainly shaped by dispersal limitation, while homogeneous selection dominates rare fungal assembly (Jiao and Lu, 2020a). More importantly, variations in environmental factors can alter the balance between different assembly processes (Dini-Andreote et al., 2015; Tripathi et al., 2018). Depending on ecosystem types and inquiry scales, the community assembly of soil microbes is influenced by various environmental variables such as aridity, temperature and soil attributes (Zhang K. et al., 2019; Delgado-Baquerizo et al., 2020; Jiao and Lu, 2020b). In deserts, soil microbes are deeply challenged by non-living stressors such as drought, high temperature and nutrient-limitation (Alsharif et al., 2020). And these stressors, especially drought stress, have been found to govern abundant and rare bacterial assembly (Wang et al., 2021b). However, how multiple abiotic stressors jointly drive the assembly processes of abundant and rare fungi across large-scale deserts remains unclear.

In addition to abiotic factors, plant communities have been broadly documented as critical factors regulating soil microbial assembly (Ning et al., 2020; Liu L. et al., 2021). Plant-microbial interactions forcefully influence, and are affected by, biodiversity and ecosystem processes (Wardle, 2006; van Dam and Heil, 2011; Wagg et al., 2014). Plant communities drive microbial assembly through host specificity, promoting resource partitioning and niche differentiation (Wardle et al., 2004; Gould et al., 2016; Pei et al., 2016). Desert vegetation is characterized by discrete plant patches, where water and soil nutrients are captured and accumulated under the canopies, and thus greater biological activities are observed compared with the situation in adjacent areas (Okin et al., 2004; De Graaff et al., 2014), leading to the formation of fertile islands (Ochoa-Hueso et al., 2018). Therefore, we posited that plant communities would play key roles in mediating the assembly processes of soil fungi in deserts. However, little is known about the internal effects of plant communities on the assembly processes of abundant and rare fungi across deserts.

In this study, we conducted a 2,500-km regional transect survey along regional climatic, edaphic and vegetational gradients across deserts in China. We collected 90 desert soil samples and then assessed soil fungal communities by high-throughput sequencing approaches. We aimed to examine three hypotheses: (1) Differential assembly mechanisms shape the distribution patterns of abundant and rare fungi across deserts; (2) Multiple abiotic stressors jointly mediate the assembly processes of soil fungal communities, while their roles vary between rare and abundant subcommunities; (3) Plant communities play key roles in regulating the assembly processes of rare and abundant fungal sub-communities.

## Materials and methods

### Data collection

In 2018, a total of 30 sites were randomly selected across major climatic zones (Hyper arid and arid zones) and vegetation type, including 14 for shrubby desert, 9 for semi-shrubby and dwarf semi-shrubby desert, 4 for shrubby steppe desert, 3 for dwarf semi-arboreous desert. These 30 sites encompass a wide environmental gradient (mean annual temperature ranging from 0.9 to 10.4°C, aridity index ranged from 0.03 to 0.32) and altitudinal gradient (altitude ranged from 849 to 2,391 m).

At each site, along a 1-km transect, we randomly selected three 10 × 10 m quadrats spaced at approximately 300-m intervals from the representative vegetation. For each quadrat, plant cover (PLC), height (PHight) and crown diameter (Cwidth), abundance of each species were measured and recorded. Plant functional traits, including specific leaf area (SLA), leaf nitrogen: phosphorus ratios (LNP), leaf carbon: nitrogen ratios (LCN) were measured at each plot with procedures described by previous studies (Cornelissen et al., 2003; Wang et al., 2016). Both intraspecific and interspecific variations in each functional trait were incorporated simultaneously. All functional traits were transformed to community weighted means, and CWM value was used to represent the functional trait of a community.

In each quadrat, a total of 20–30 bulk soil cores (10 cm depth) were randomly sampled under the canopy of the plants and subsequently mixed together into a composite sample. We used a 2-mm mesh to sieve all composite samples, and subsequently divided those samples into two portions. One portion was stored at 4°C to assess soil property, and the other was stored at –20°C until DNA extraction. Together, a total of 90 soil samples from 30 sites were used in this study. Six soil variables, soil moisture (SM), pH, total organic carbon (TOC), total phosphorus (TSP), available nitrogen (SAN) and total nitrogen content (TSN) were also measured as described by Wang et al. (2020).

Mean annual temperature (MAT) and mean annual precipitation (MAP) were obtained from WorldClim database. Annual potential evapotranspiration (PET) data were extracted from CGIAR-CSI. We calculated aridity level [1- aridity index (AI)], where AI was defined as the ratio of MAP to potential evapotranspiration (that is, AI = MAP/PET). Finally, MAT and aridity were selected to represent temperature and drought stress, respectively.

### Molecular analysis

Total fungal DNA were extracted from 0.5 g of well-mixed fresh soil samples using E.Z.N.A. soil DNA kits (OMEGA, USA) following the manufacturer’s instructions. The fungal internal transcribed spacer (ITS) region was amplified using the universal primers ITS1F (5′-CTTGGTCATTTAGAGGAAGTAA-3′) and ITS2 (5′- TGCGTTCTTCATCGATGC-3′). PCR reactions were conducted in triplicate for each sample. The PCR program was as follows: 94°C for 5 min, 25 cycles at 94°C for 30 s, 55°C for 30 s, and 72°C for 30 s with a final extension of 72°C for 10 min. PCR amplicons were extracted from 2% agarose gels and purified using the AxyPrep DNA Gel Extraction Kit (Axygen Biosciences, Union City, CA, USA) according to the manufacturer’s instructions and quantified using QuantiFluor™ –ST (Promega, USA) Purified amplicons were pooled in equimolar and paired-end sequenced (2 × 300) on an Illumina Miseq PE300 sequencing platform at Beijing Allwegene Tech, Ltd. (Beijing, China).

Raw sequences were processed by the ASV method within the QIIME package (Version 1.8). Fungal sequences <200 bp or with an average quality score < 20 were removed. After that, the filtered sequences were denoised by using DADA3. Notably, ASVs with reads less than 8 were discarded to avoid the random influence of identification of rare taxa. This methodology was widely used in previous studies on abundant and rare microbes (Liu et al., 2015; Gao et al., 2020). To eliminate for influence of different sequencing depth on the analyses, each sample was randomly rarefied to 9,080 sequences (minimum) for subsequent analyses. Soil fungal raw sequences used in this paper are available in the NCBI Sequence Read Archive under BioProject PRJNA722881.

### Definition of abundant and rare fungi

The combination of regional (across all samples) and local (one sample) relative abundances was used to define rare and abundant fungi according to the classification criteria used in previous studies (Liu et al., 2015; Jiang et al., 2019). Specifically, ASVs with relative abundances <0.01 and >1% in a single sample were regarded as locally rare and abundant, respectively (Pedrós-Alió, 2012; Liu et al., 2015). Afterward, the average relative abundances of specific OTUs in all samples were assessed. Given that a great number of ASVs with extremely low abundances were removed, specific ASVs with average relative abundances >0.1% in all samples were defined as regionally abundant (Logares et al., 2014), whereas those with average relative abundances <0.005% in all samples were considered as regionally rare. Regional abundant and rare ASVs were used in subsequent analysis. Additionally, abundant and rare ASVs were classified into different functional groups using FUNGuild. Propagule size was considered as the body size of each abundant and rare species, and was identified using the methods of previous studies (Zinger et al., 2019; Luan et al., 2020).

### Statistical analyses

Eight abiotic variables (climate: Aridity and MAT; soil attributes: SM, pH, TSN, AN, TSP, and TOC) and eight plant-attribute variables (PLC, SLA, LCN, LNP, PHight, Cwidth, plant richness, and community composition) were used in this study. When necessary, environmental data would be log-transformed before the analyses. To reduce the strong collinearity among variables, we removed MAT, TSN, TOC, and plant richness because of the higher correlation between those variables (i.e., Pearson’s *r* > 0.6; Supplementary Figure 1). After that, Bray-Curtis dissimilarity distance was estimated to reflect the variance in species composition among plant communities. Geographic distance matrices were calculated based on GPS coordinates, and then we calculated standardized environmental Euclidean distance within “vegan” package.

Levins’ niche breadth (B) index was employed to elucidate the patterns of stochastic and deterministic processes and their effects on soil fungal communities (Levins, 1968). The *B* value of each fungal OTU was calculated following the previous method of Jiao and Lu (2020a). A higher B value indicates a wider habitat niche breadth. Community *B*-values (Bcom) were quantified by abundance-weighted mean *B*-values from all fungal OTUs occurring within each community (Wu et al., 2018). A fungal community with a higher *B* value is expected to be more metabolically flexible (Pandit et al., 2009; Wu et al., 2018). Notably, the “niche.width” function of the “spaa” R package was applied to calculate Levins’ niche breadth (B) index.

Abundance-based null model and neutral model analyses were used to infer the influence of ecological processes on soil fungal assembly (Kraft et al., 2011; Myers et al., 2013; Ning et al., 2019). In brief, 999 null local communities were generated through randomly resampling individuals into a local community with probabilities proportional to the regional abundance of the species whereas maintaining same species richness and abundance (Ning et al., 2019; Liu W. et al., 2021). Afterward, the standardized effect size (β-deviation) of β-diversity was calculated using the following formula: β-deviation = (β-diversity_*obs*_ – Mean(β-diversity_*null*_))/standard deviation (β-diversity_*null*_), where β-diversity_*null*_ and β-diversity_*obs*_ can denote the mean Bray–Curtis dissimilarity of null communities and observed β-diversity, respectively. Community assembly is dominated by stochastic processes if the β-deviation is statistically indistinguishable from zero; otherwise, the β-deviation remarkably greater than zero indicates a dominant influence of dispersal limitation or heterogeneous selection. Conversely, the domination of homogenizing dispersal or homogeneous selection would be supported if the β-deviation is significantly less than zero (Zhang et al., 2020). The null-model approach conducted based on phylogenetic β-diversity can better evaluate the relative roles of these processes (Stegen et al., 2013). However, fungal ITS is a variable region and cannot be aligned, so this study did not implement such analyses (Zinger et al., 2019). Meanwhile, the contribution of stochastic processes was further calculated using neutral model via forecasting the association between abundance and frequency of taxonomic occurrence (Sloan et al., 2006). *R*^2^ indicates the fitness of the neutral model.

To further identify the relative strength of dispersal limitation and environmental selection, we partitioned the relative effects of space and environment on β-deviations through variation-partitioning analysis (VPA) based on multiple-regression matrices (MRMs) (Goslee and Urban, 2007). In MRM test, all factors were subjected to forward selection until *P* < 0.05. Then, VPAs were performed using previous methods (Peres-Neto et al., 2006). The individual influence of spatial factors represents the effect of dispersal limitation, whereas the individual effect of environmental distance indicates the importance of environmental selection (Myers et al., 2013; Zhang et al., 2020). After that, hierarchical partitioning analysis was applied to quantified the relative contribution of plant attributes, soil and climatic factors in the final model of MRM. The slope of ordinary least-square regression between compositional similarity (1- β-diversity) and geographic distance was further used to quantify the distance–decay relationships (DDRs).

To precisely elucidate how abiotic and biotic factors jointly drive the variation in the relative importance of different assembly processes, structural-equation models (SEMs) were established in this study. Before the analysis, priori SEMs were established based on the related theoretical hypothesis (Supplementary Figure 2). χ^2^-test, comparative fit index, goodness of fit index, low root-square-mean error of approximation were used to test the fitness of SEMs. SEM was conducted within the “lavaan” package.

## Results

### Taxonomic and functional composition of abundant and rare fungi

A total of 819,966 soil fungal high-quality sequences were identified from 90 samples, which were clustered into 4,082 fungal ASVs. Among them, 2,755 ASVs were classified as rare fungi, which only accounted for 4.14% of the total sequences (Supplementary Table 1). In contrast, only 160 ASVs were classified as abundant fungi, whereas their total relative abundance accounted for 70.72% of the entire community. Wilcoxon rank-sum test revealed that abundant fungi had a significantly larger body size than the rare (Supplementary Figure 3). Furthermore, the relative abundances of both abundant and rare fungi were not related to longitude (Supplementary Figures 4A,B). The relative abundances of abundant fungi were higher at medium latitude than at two ends of the latitude gradient, whereas those of rare fungi showed an opposite trend (Supplementary Figures 4C,D).

**FIGURE 1:**
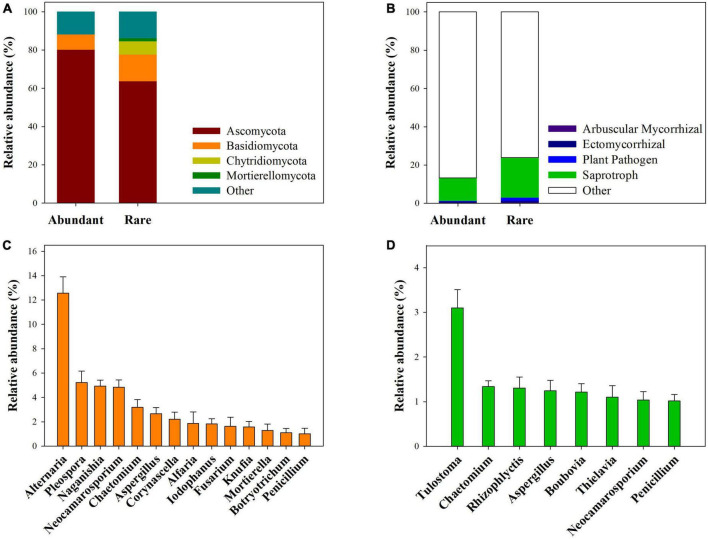
Taxonomic and functional composition of abundant and rare fungal sub-communities. **(A)** Showed dominant phylum and their total relative abundance for the abundant and rare fungal subcommunities. **(B)** Showed total relative abundance of Saprotrophic, Arbuscular Mycorrhizal, Ectomycorrhizal, and Plant-pathogen fungal. **(C,D)** Showed the dominant genera and their average relative abundance for the abundant and rare fungal subcommunities across 90 samples.

At the phyla level, abundant subcommunities were mainly dominated by *Ascomycota* (80.30%) and *Basidiomycota* (7.97%), while rare subcommunities were mainly dominated by *Ascomycota* (63.90%), *Basidiomycota* (13.87%), *Chytridiomycota* (6.96%), and *Mortierellomycota* (1.57%) (Figure 1A). At the genera level, abundant subcommunities were mainly dominated by *Alternaria*, *Pleospora*, *Naganishia*, *Neocamarosporium*, *Chaetomium*, *Aspergillus*, *Corynascella*, *Alfaria*, *Iodophanus*, *Fusarium*, *Knufia*, *Mortierella*, *Botryotrichum*, and *Penicillium* (Figure 1C), and rare subcommunities were mainly dominated by *Tulostoma*, *Chaetomium*, *Rhizophlyctis*, *Aspergillus*, *Boubovia*, *Thielavia*, *Neocamarosporium*, and *Penicillium* (Figure 1D). Among these dominant genera, the relative abundance of *Neocamarosporium*, *Mortierella*, and *Botryotrichum* for abundant fungi, and that of *Tulostoma* for rare fungi significantly differed across four vegetation types (Supplementary Figures 5, 6). Furthermore, saprotrophic, arbuscular mycorrhizal, ectomycorrhizal and plant pathogen together accounted for 13.25 and 23.97% of the sequences of abundant and rare fungi, respectively (Figure 1B).

### Site occupied and habitat niche breadth

**FIGURE 2:**
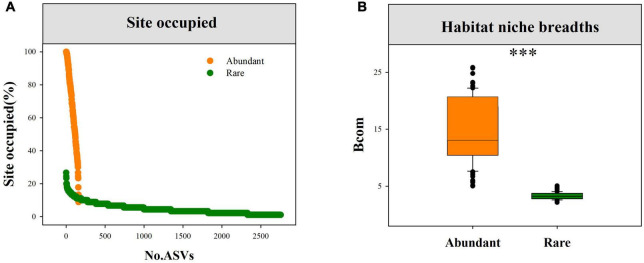
The proportion of sites occupied for each abundant and rare fungi **(A)**, and the difference in mean habitat niche breadths (Bcom) between abundant and rare fungal subcommunities **(B)**, and ^***^P < 0.0001; Wilcoxon rank-sum test.

Among, 160 abundant ASVs, 114 ASVs (71.25%) were found in more than 50% of the samples (Figure 2A). In contrast, only 3 rare ASVs (0.11%) were found in more than 20% of the sites. In addition, community-level niche breadths (Bcom) were employed to elucidate the relative importance of deterministic and stochastic processes in fungal community assembly. As expected, we observed remarkably higher mean Bcom values in abundant fungal subcommunities than in rare subcommunities (Figure 2B).

### Assembly processes of rare and abundant fungal subcommunities

**FIGURE 3:**
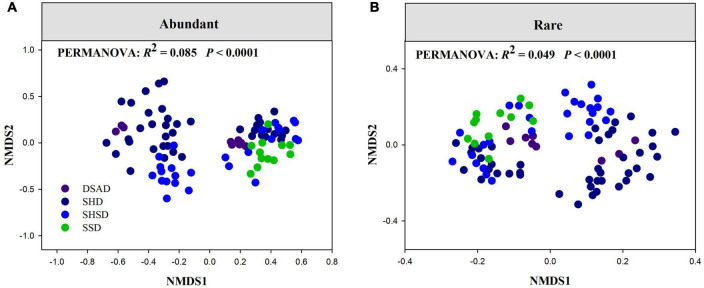
Non-metric multidimensional scaling (NMDS) ordination of the community composition of **(A)** abundant and **(B)** rare fungi across four vegetation types. DSAD, Dwarf Semi-arboreous Desert; HSPD, Shrubby Steppe Desert; SHD, Shrubby Desert; SHSD, Semi-shrubby and dwarf semi-shrubby desert.

There were significant distance-decay relationships (DDRs) between spatial distances and community similarity (1 – observed β-diversity) in abundant and rare fungal subcommunities (*P* < 0.0001, Supplementary Figure 7A). Additionally, the slope of DDRs estimated by least squares regression models for both abundant and rare fungal subcommunities was meaningfully less than zero (*P* < 0.0001), but abundant fungal subcommunities had a faster species turnover rate (slope = –0.037) than rare fungal subcommunities (slope = –0.011). Meanwhile, rare fungal subcommunities had remarkably higher observed β-diversity values than abundant fungal subcommunities (*P* < 0.001, Supplementary Figure 7B). Permutational analysis of variance (PERMANOVA) demonstrated that the community composition of abundant and rare fungi significantly varied across four vegetation types (*P* < 0.001, Figures 3A,B). MRM revealed that the species composition of both rare and abundant fungi was prominently determined by space, SM and plant composition (*R*^2^ = 0.26 and 0.27, respectively; Supplementary Table 2).

**FIGURE 4:**
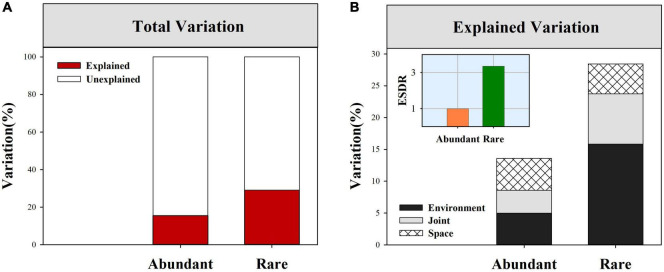
Percent of β-deviations for abundant and rare fungal subcommunities explained by spatial and environmental variables. **(A)** Total variation in β-deviations were explained by full model. **(B)** Variation in β-deviations were explained by spatial and environmental variables after forward-model selection.

Neutral community models exhibited a lower model fit in rare sub-communities (*R*^2^ = 0.26) than in abundant sub-communities (*R*^2^ = 0.91, Table 1). Null model analysis showed that β-deviations for both abundant and rare fungal subcommunities were significantly greater than zero (Supplementary Figure 8), implying the dominance of dispersal limitation or heterogeneous selection. VPA showed that environment and space together explained a larger proportion of the variation in rare fungal β-deviations than in the abundant (Figure 4A). Moreover, environment and space could individually explain a proportion of the variation in both abundant and rare fungal β-deviations, but environment had a more important influence on rare fungal assembly (Figure 4B). Specifically, space individually explained 5.02 and 4.71% of the variation in abundant and rare fungal β-deviations, respectively. Environmental factors individually explained 5.0 and 15.83% of the variation in abundant and rare fungal β-deviations, respectively. Finally, we found that the unexplained component of fungal β-deviations was larger in abundant subcommunities than in the rare.

**TABLE 1:** Fit of the neutral model in abundant and rare fungal sub-communities in desert soil.

	*m*	*R* ^2^
Abundant	0.45	0.91
Rare	0.50	0.26

### Effects of plant attributes, soil and climatic factors on the assembly processes of rare and abundant fungal subcommunities

**FIGURE 5:**
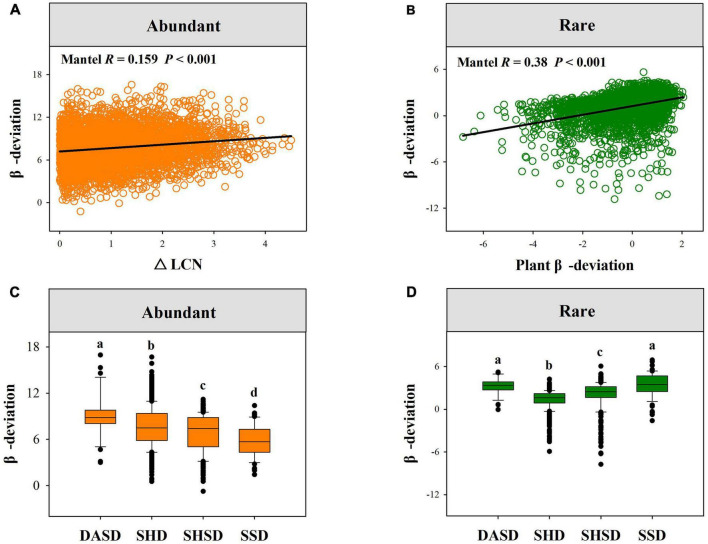
The relationships between fungal β-deviation and difference in plant attributes. **(A)** Showed the relationships between abundant fungal β-deviation and difference in LCN. **(B)** Showed the relationships between rare fungal and plant β-deviation. **(C,D)** Showed the variation in the β-deviation of abundant and rare fungi across four vegetation types. SSD, shrubby steppe desert; SHSD, semi-shrubby and dwarf semi-shrubby desert; SHD, shrubby desert; DSAD, dwarf semi-arboreous desert.

Mantel tests showed that both rare and abundant fungal β-deviations were significantly related to plant-related, soil and climatic variables (Supplementary Table 3). We observed that the β-deviation values of both abundant and rare fungi were positively related to plant β-deviations, and the divergence in soil, climatic and plant traits (Figures 5A,B and Supplementary Table 3). More importantly, the β-deviations of rare and abundant fungi significantly varied across four vegetation types (Figures 5C,D).

**FIGURE 6:**
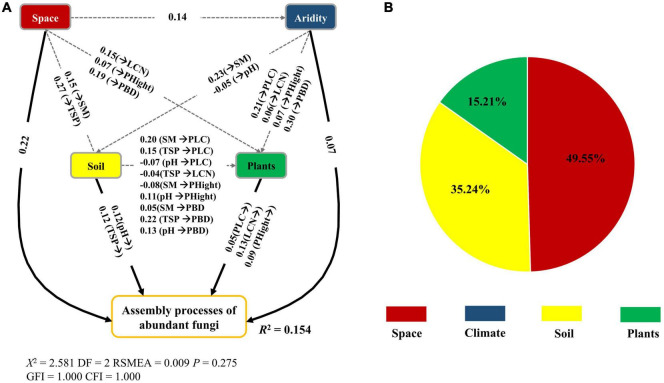
Direct and indirect effect of spatial and environmental variable on the assembly processes of abundant fungi **(A)**, and the relative influence of spatial, abiotic (soil and climate), and plant attributes on the assembly processes of abundant fungi **(B)**. Only significant links were reported (*P* < 0.05). Space, spatial variables.

**FIGURE 7:**
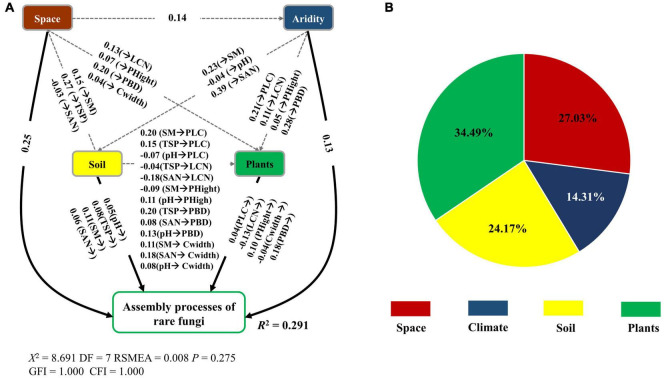
Direct and indirect effect of spatial and environmental variable on the assembly processes of rare fungi **(A)**, and the relative influence of spatial, abiotic (soil and climate), and plant attributes on the assembly processes of rare fungi **(B)**. Only significant links were reported (*P* < 0.05). Space, spatial variables; PBD, plant β-deviation.

The fitted SEM models showed that space, plant attributes, abiotic (soil and climate) factors together explained 15.40 and 29.10% of the variation in the β-deviations of abundant and rare fungi, respectively (Figures 6A, 7A). Spatial, soil and climatic factors had a direct and indirect influence on abundant and rare fungal β-deviations. Plant functional traits and species composition significantly influenced rare fungal β-deviations, whereas only plant functional traits had a direct effect on abundant fungal β-deviations. Hierarchical partitioning analysis indicated that abundant fungal β-deviations were mainly regulated by space, followed by soil factors and plant attributes. However, rare fungal β-deviations were predominantly driven by plant attributes, followed by plant attributes, space, soil and climatic factors (Figures 6B, 7B). Moreover, rare fungal β-deviations were more influenced by plant attributes than the abundant.

## Discussion

### Differential assembly mechanisms lead to distinct distribution patterns of abundant and rare fungi in deserts

In this study, we observed that abundant and rare fungal sub-communities were dominated by different genera, and more saprotrophic, arbuscular mycorrhizal, ectomycorrhizal and plant-pathogen fungi were detected in rare subcommunities than in the abundant, revealing that abundant and rare fungal sub-communities were dominated by different species with distinct functional traits, respectively. Meanwhile, the slope of DDR in rare fungal subcommunities was meaningfully steeper than that in abundant subcommunities, which is similar to the findings of a previous study on desert bacteria (Wang et al., 2021b). In our study, abundant fungi were more ubiquitous than rare taxa, implying that rare fungi were govern by the presence of distinct ecological niches (Jiao et al., 2017). Therefore, rare sub-communities were heavily limited by local habitat specificity (Barberán et al., 2014), thereby leading to a higher species turnover rate. In addition, abundant and rare fungi showed different shifts in abundance along latitudinal gradients. These results reveal that rare and abundant fungi exhibit differential distribution patterns.

Elucidating the difference in assembly processes between abundant and rare microbes is crucial to unraveling the mechanisms underlying global change in soil biodiversity (Nemergut et al., 2013; Lynch and Neufeld, 2015; Jia et al., 2018). In our study, neutral model analysis revealed that abundant sub-communities were more influenced by neutral processes than the rare. Null model analysis and VPA demonstrated that heterogeneous selection and dispersal limitation worked together to govern both abundant and rare fungal assembly, which is similar to the findings on entire fungal communities (Wang et al., 2017). Our results further indicated that dispersal limitation had an important but equal influence on both abundant and rare sub-communities. We observed that a majority of rare species occurred in less than 10% of the sampling sites, which might lead to rare fungi are easily influenced by dispersal limitations (Jiao and Lu, 2020a). On the other hand, abundant fungi with a larger body size may be more easily influenced by dispersal limitations (De Bie et al., 2012). Therefore, both abundant and rare fungal assembly was influenced by dispersal limitations. Notably, our finding was inconsistent with previous findings that abundant microbes are more limited than rare microbes by dispersal (Liu et al., 2015; Wu et al., 2017; Jiao and Lu, 2020a). This discrepancy may be attributed to the difference in environmental regimes and spatial scales among different studies (Liu et al., 2015).

Notably, dispersal limitation and heterogeneous selection played almost equally important roles in abundant fungal assembly. However, heterogeneous selection rather than dispersal limitation played a dominated role in rare fungal assembly. Additionally, rare fungal assembly was more strongly affected by heterogeneous selection than the abundant. Functional traits may mediate species abundance and assembly processes along environmental gradients by affecting their fitness and performance (McGill et al., 2006; Webb et al., 2010). Compared with rare species, abundant species are expected to have optimal traits in desert environments, thereby adjusting themselves to stressful environments (Brown, 1984; Umaña et al., 2015). In this study, abundant fungi exhibited a wider habitat niche breadth and were distributed more ubiquitously than rare fungi, indicating that abundant fungi occupy diverse niches, and have higher resource competitiveness and greater tolerance to abiotic stresses than rare fungi (Jiao et al., 2017). Therefore, rare fungal assembly was more affected by environmental selection. Notably, the pure influence of spatial factors also potentially reflects the effects of other underlying mechanisms that occur in parallel with dispersal limitation (Bell, 2010; Martiny et al., 2011). For example, historical processes, could also lead to the difference in fungal community composition (Dexter et al., 2012). The unexplained component of community variation has often been interpreted as resulting from local stochastic demographic processes (De Cáceres et al., 2012). In consequence, we suggest that difference in the relative role of heterogeneous selection, dispersal limitations and other, unknown processes in abundant and rare sub-communities lead to the differential biogeographic patterns of abundant and rare fungi.

### Plant communities and soil factors drive the community assembly processes of rare abundant fungi in deserts

Uncovering ecological drivers governing community assembly is a central issue in ecology (Tripathi et al., 2018). However, there is a knowledge gap in how environmental factors regulate the relative strength of different processes in mediating the community assembly of abundant and rare fungi in desert soils. In general, our results indicated that the β-deviations of both abundant and rare fungal subcommunities were mainly determined by spatial and environmental variables together. Traditional studies on dryland ecosystems believe that climatic factors, especially aridity, determine soil microbial community assembly (Maestre et al., 2015; Delgado-Baquerizo et al., 2020). However, SEM and hierarchical partitioning analysis showed that the assembly processes of rare and abundant fungi were more influenced by plant communities and soil factors rather than climate. This is consistent with the result of a previous report on dryland plant community assembly (Wang et al., 2019). There are several interpretations for the results presented here. In desert habitat, soil water and nutrients resources might be strongly redistributed by microtopography and discrete plant communities. This would further heighten the spatial heterogeneity of water and nutrients supply (Reisner et al., 2013). Hence, plant communities and soil condition could play dominant roles in governing soil fungal assembly by multiple processes, such as recruitment limitation (Grubb, 1977) and resource competition (Stevens and Carson, 2002). It is also notable that climatic factors, such as aridity, play fundamental roles in nitrogen cycling of dryland ecosystems (Fernandez-Going et al., 2013; Wang et al., 2014). Hence, climatic factors can indirectly drive soil fungal assembly via influencing soil conditions and plant communities, as shown in SEM results. Additionally, strong couplings among climatic, soil and plant-related factors make it difficult to quantify their internal effect precisely.

Numerous studies have revealed that plant communities strongly affect soil fungal species richness and composition (Yang et al., 2017; Wang et al., 2018). In agreement with the findings on desert soil bacteria (Wang et al., 2021b), we found that both plant functional traits and composition could significantly regulate the assembly processes of both abundant and rare fungi. Particularly, plant functional traits together with plant composition provided a better prediction of abundant fungal assembly than spatial and abiotic factors, which is consistent with a previous study (Liu L. et al., 2021). There are several interpretations of the key role of plant communities in regulating the assembly processes of soil fungi. First, as a key determinant of desert ecosystem structure and functioning (Whitford and Duval, 2019), the formation of “fertile islands” is mainly driven by plant community structure and traits (Ochoa-Hueso et al., 2018). Hence, plant attributes can effectively mediate water and nutrient availability by influencing the formation of fertile islands (Okin et al., 2004; De Graaff et al., 2014), in turn affecting soil fungal assembly processes. Second, fungi are known to interact closely with plants in dryland ecosystems (Collins et al., 2008; Pointing and Belnap, 2012). Plant-microbe interactions can affect microbial fitness and performances (Maestre et al., 2009; Martínez-García et al., 2015), thus in turn mediating soil fungal assembly (Delgado-Baquerizo et al., 2018). Meanwhile, the relative strength of different assembly processes varied across four vegetation types, which may be partly due to the difference in environmental conditions and spatial scales. For example, almost all climatic, soil and plant-related variables show significant group differences across four vegetation types (Wang et al., 2020).

### Stronger influence of environmental factors on rare fungal assembly than on the abundant

Previous studies have widely observed the different response of abundant and rare species to environmental factors (Jiao and Lu, 2020a; Liang et al., 2020; Wang et al., 2021b,^2022^). In this study, we found that both soil and climatic factors exhibited stronger effect on the assembly processes of rare fungi than the abundant. Compared with rare species, abundant species have wider niche breadth and can competitively utilize an array of resources and effectively adapt to the environment (Jiao et al., 2017). Hence, rare fungal assembly might be more sensitive and vulnerable to the variation in environmental condition. Notably, we also found that the assembly process of rare fungi was more influenced by plant attributes than the abundant. It is explicitly known that fungi can closely interact with plants in dryland ecosystems (Collins et al., 2008; Pointing and Belnap, 2012). In fact, we detected more saprotrophic, mycorrhizal and plant-pathogen fungi in rare subcommunities than in the abundant. Hence, plant functional traits and composition can generate a powerful influence on rare fungal assembly by promoting resource partitioning and niche differentiation (Wardle et al., 2004; Gould et al., 2016), as well as by facilitating antagonistic and mutualism interactions with soil fungi (Delgado-Baquerizo et al., 2018). Additionally, abundant fungi occupy more diverse niches, and have higher resource competitiveness, as well as greater adaptability to the biotic environment (Jiao et al., 2017). Therefore, it is difficult for plant communities to effectively affect abundant fungal assembly by altering abiotic and biotic attributes.

## Conclusion

This study conducted a comprehensive comparison of the biogeographical patterns and assembly mechanisms between abundant and rare fungi across desert ecosystems, and summarized how environmental factors drove the assembly processes of abundant and rare fungi. We observed that abundant fungi exhibited wider niche breadth and greater presence than the rare. Neutral processes played a more important role in shaping abundant sub-communities compared to the rare sub-communities. Our results further demonstrated that the relative effects of stochastic and deterministic processes varied between abundant and rare sub-communities. Plant functional traits and composition plays a critical but different role in driving community assembly of both abundant and rare fungi. These findings could contribute to infer the mechanisms underlying the generation and maintenance of soil fungal diversity in harsh drylands.

## Data availability statement

The datasets presented in this study can be found in online repositories. The names of the repository/repositories and accession number(s) can be found in the article/Supplementary material.

## Author contributions

JW and JL designed the study. JW and YW performed the field investigation and collected the data. JW and NH developed the methods. JW wrote the manuscript. MQ and YW helped with the data analysis. MQ and NH conducted the language editing. All authors discussed the results and contributed significantly to the final manuscript.
