# Supplementary material for: Plant traits and community composition drive the assembly processes of abundant and rare fungi across deserts
Source: Front Microbiol. 2022 Sep 28;13:996305. doi: 10.3389/fmicb.2022.996305 (PMC9554466; doi:10.3389/fmicb.2022.996305)
Supplement: Supplementary file 1 [file Data_Sheet_1.doc]

**Plant traits and community composition drive the assembly processes of abundant and rare fungi across deserts**

Jianming Wang^1^, Yin Wang^1^, Mengjun Qu**^1^**, Nianpeng He**^2^**, Jingwen Li^1^*

1. School of Ecology Nature Conservation, Beijing Forestry University, Beijing, China.

2. Key Laboratory of Ecosystem Network Observation and Modeling, Institute of Geographic Sciences and Natural Resources Research, Chinese Academy of Sciences, Beijing, China

* Corresponding author

Table S1 General description of all, abundant and rare OTUs data sets

|  | OTU numbers | Sequence numbers |
| --- | --- | --- |
| All OTUs | 4,082 | 819,966 |
| Abundant OTUs | 160 (3.92%) | 579,861(70.72%) |
| Rare OTUs | 2,755 (67.49%) | 33,907 (4.14%) |

Abundant OTUs were deﬁned as the OTUs with an abundance > 1% in a sample and a mean relative abundance of > 0.1% in all samples.

Rare OTUs were deﬁned as the OTUs with an abundance < 0.01% in a sample and a mean relative abundance of < 0.005% in all samples.

Table S2 Stepwise multiple regressions of the observed β-diversity for abundant and rare fungal subcommunities with spatial and environment variables.

Space, spatial variables；PMS, plant compositional dissimilarity.

| **Variables** | **Abundant(*R*^2^=0.258)** | |  | **Rare(*R*^2^=0.269)** | |
| --- | --- | --- | --- | --- | --- |
|  | Coefficients (b) | *P* |  | Coefficients (b) | *P* |
| Space | 0.028 | <0.001 | Space | 0.009 | <0.001 |
| SM | 0.023 | <0.001 | SM | 0.004 | <0.001 |
| PMS | 0.119 | <0.001 | PMS | 0.023 | <0.001 |

Table S3 Mantel tests examining the associations between fungal null deviations and environmental variables

| **Variables** | **Abundant** |  |  | **Rare** |  |
| --- | --- | --- | --- | --- | --- |
|  | Mantel *R* | *P* |  | Mantel *R* | *P* |
| Spatial | 0.293 | <0.001 |  | 0.355 | <0.001 |
| Aridity | 0.129 | <0.01 |  | 0.278 | <0.001 |
| SM | 0.092 | <0.05 |  | 0.273 | <0.001 |
| SAN | 0.061 | >0.05 |  | 0.260 | <0.001 |
| TSP | 0.214 | <0.001 |  | 0.259 | <0.001 |
| pH | 0.148 | <0.01 |  | 0.114 | <0.01 |
| PLC | 0.111 | <0.05 |  | 0.180 | <0.001 |
| SLA | 0.085 | >0.05 |  | 0.018 | >0.05 |
| LCN | 0.159 | <0.001 |  | -0.093 | <0.01 |
| LNP | 0.001 | >0.05 |  | 0.016 | >0.05 |
| Cwidth | 0.055 | >0.05 |  | 0.118 | <0.001 |
| PHight | 0.111 | <0.01 |  | 0.134 | <0.01 |
| Plant β-deviation | 0.180 | <0.001 |  | 0.380 | <0.001 |

Plant β-deviation, Plant null deviations


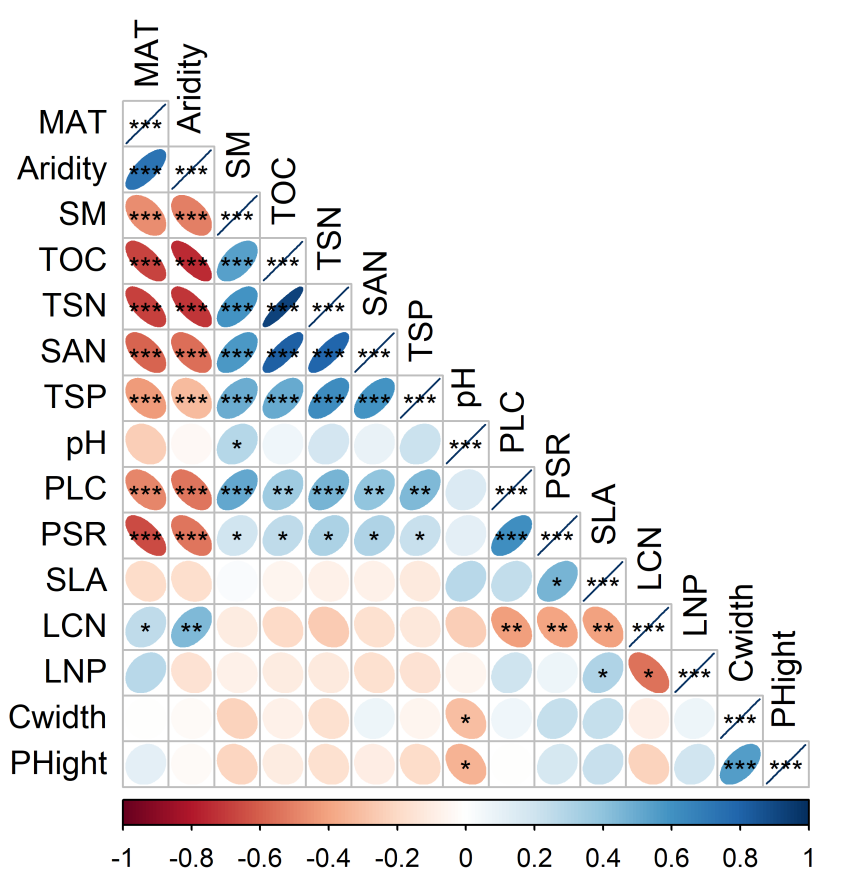


**Figure S1** Correlations among plant-related, soil and climatic variables in deserts. MAT, Mean annual temperature (℃); SM, soil moisture (%); TOC, soil total organic carbon content (g/kg); TSN, total nitrogen content (g/kg); SAN, soil available nitrogen (mg/kg); TSP, soil total phosphorus (g/kg); PLC, plant coverage (%); PSR, plant species richness; SLA, specific leaf area (cm^2^ g^-1^); LCN, Log (leaf C: N ratios); LNP, leaf N: P ratios; PHight, plant height (cm); Cwidth, canopy width (cm).


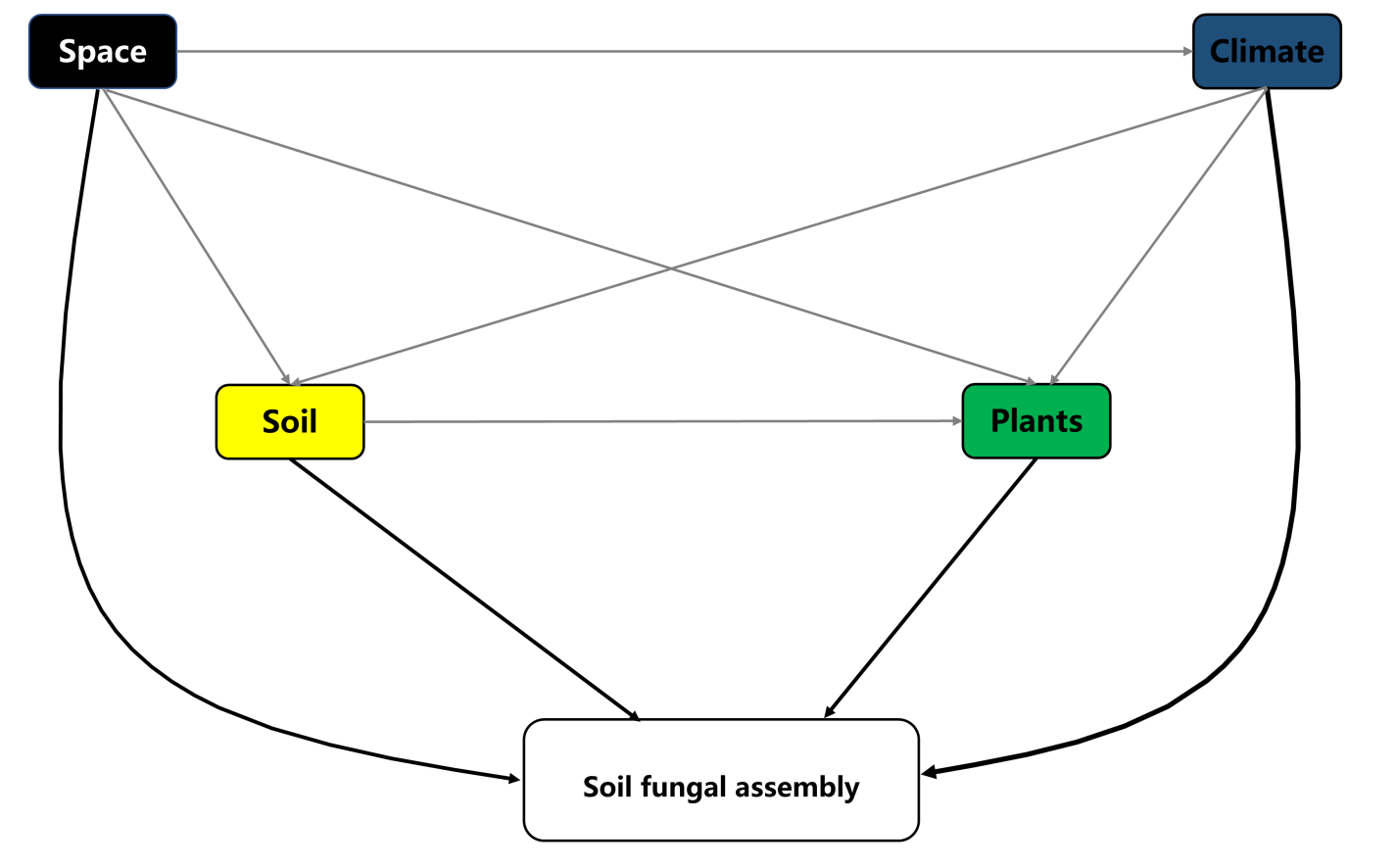


**Figure S2** Priori structural equation models including direct and indirect effects of abiotic and biotic factors on community assembly processes of soil fungi


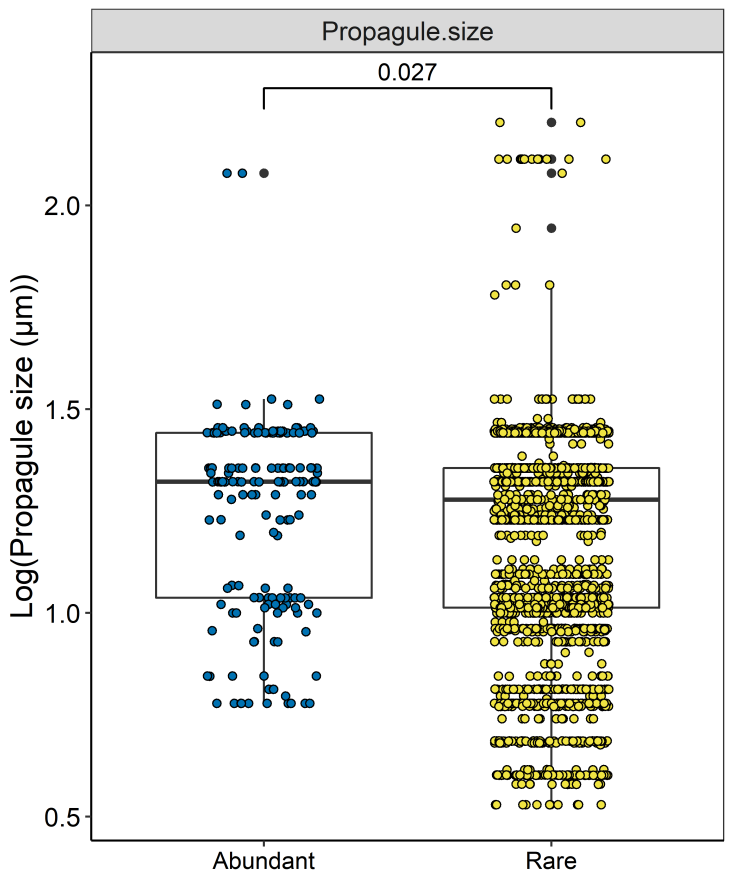


**Figure S3** Difference in body size between abundant and rare fungi


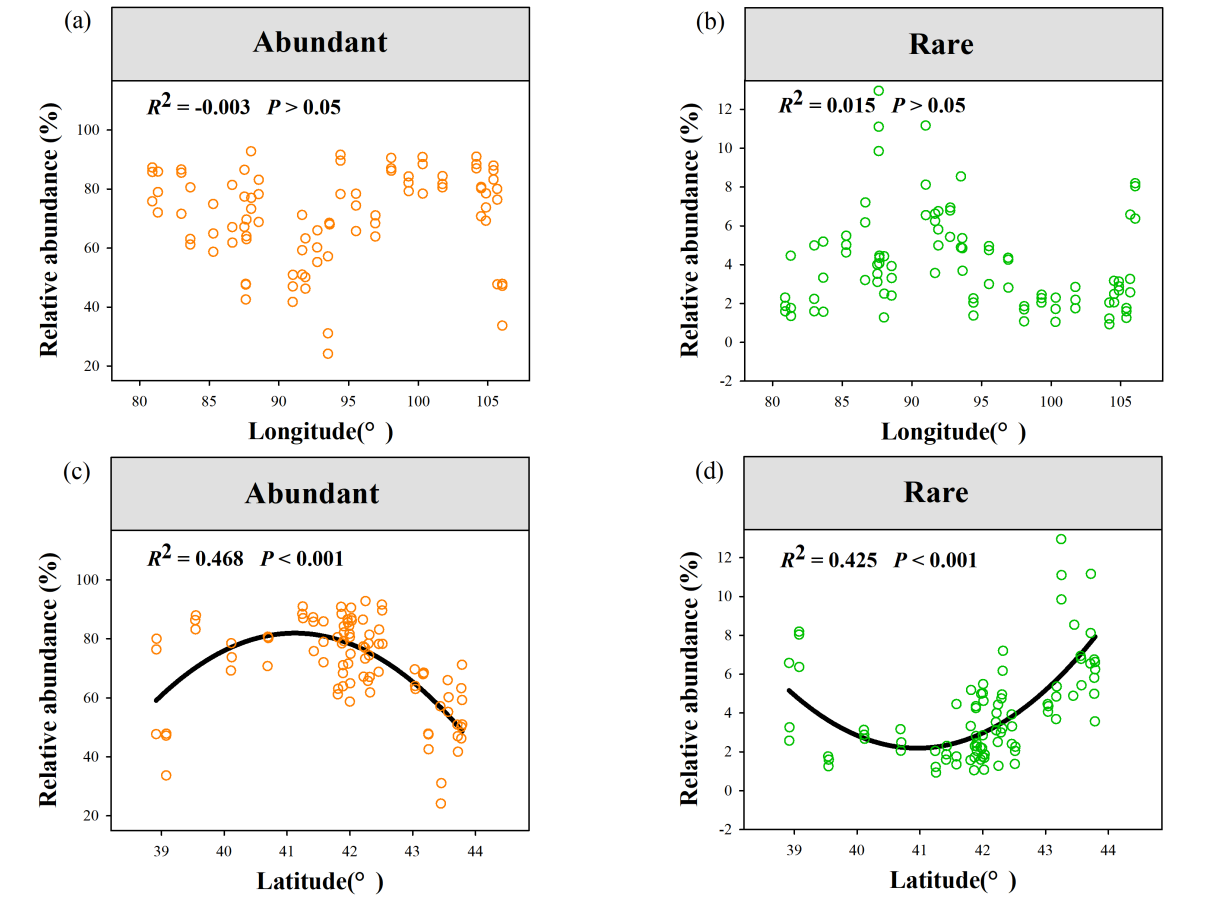


**Figure S4** Longitudinal and latitudinal patterns for relative abundance of abundant (a) and rare (b) fungi


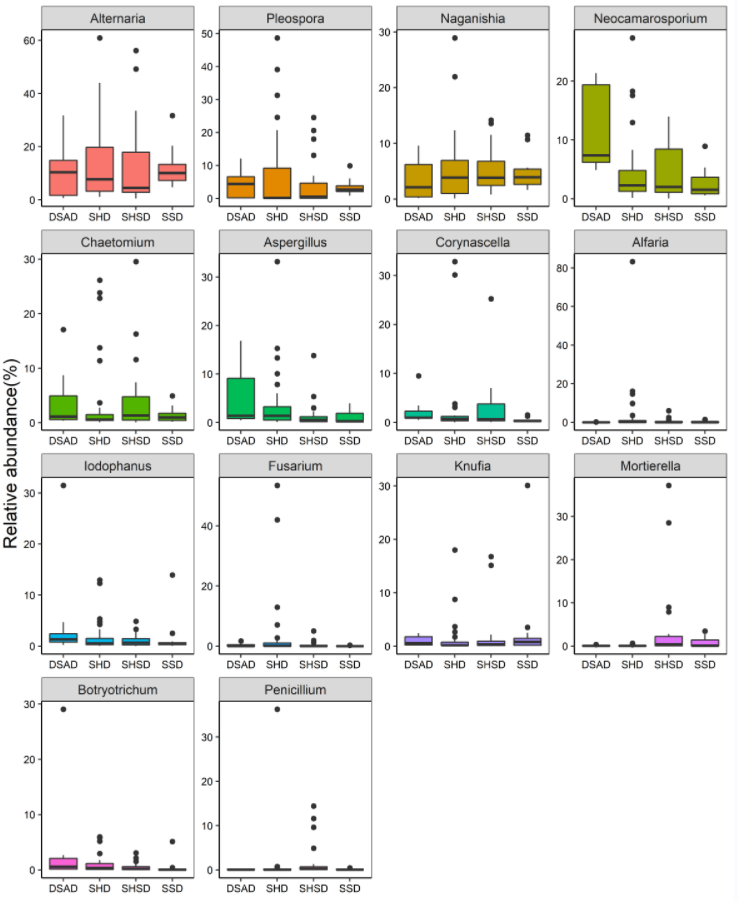


**Figure S5** Variation in the relative abundance of dominant genera for abundant fungi across different vegetation types.


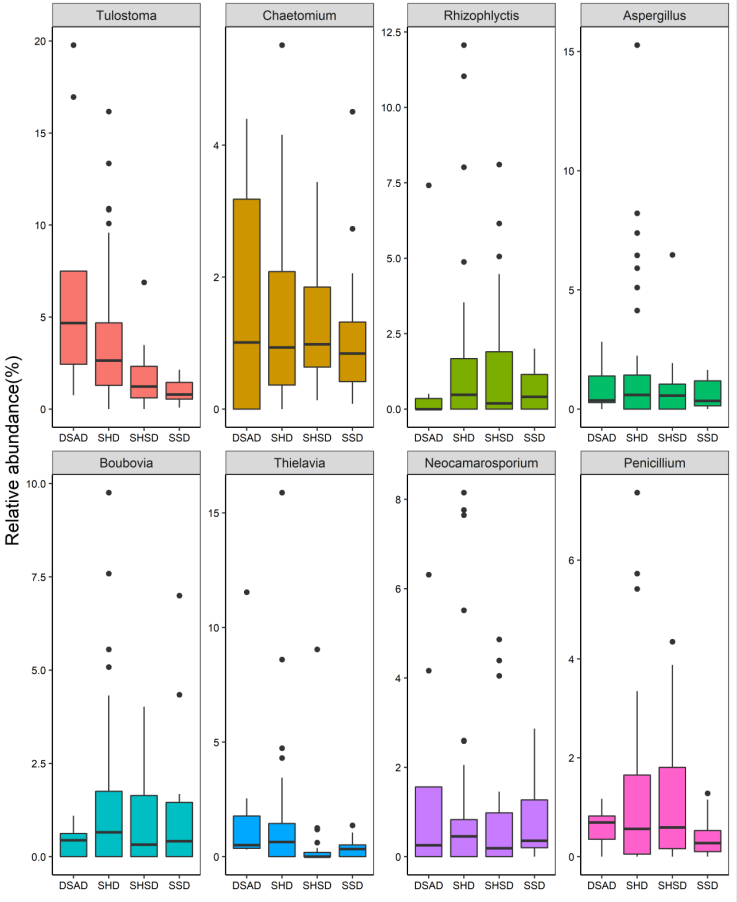


**Figure S6** Variation in the relative abundance of dominant genera for rare fungi across different vegetation types.


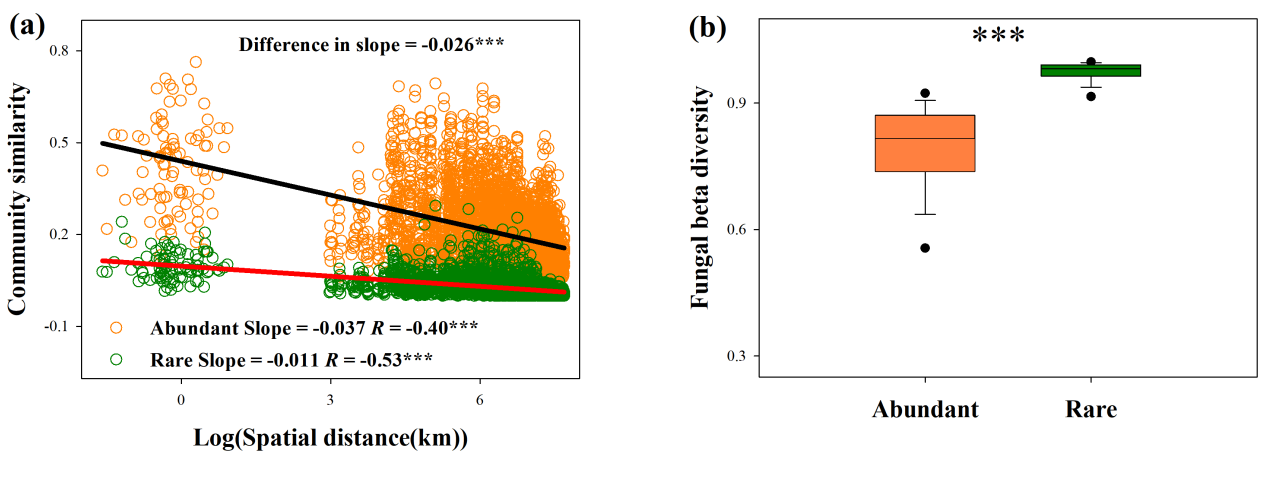


**Figure S7** Distance–decay curves of community similarity (1 – observed *β*-diversity) against spatial distances (a), and the difference in observed *β*-diversity between abundant and rare fungal subcommunities (***, *P* <0.001; Wilcoxon rank-sum test)


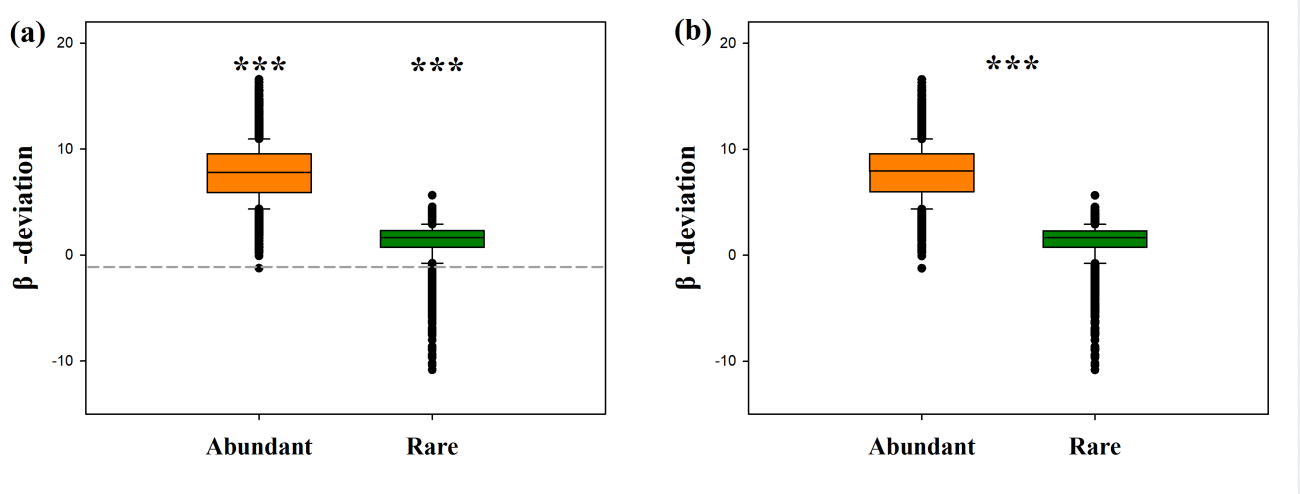


**Figure S8** Difference between abundant and rare fungal β-deviation and 0 values (a), and difference in β-deviation between abundant and rare fungal subcommunities (b). (***, *P* <0.001; Wilcoxon rank-sum test).
